# Protein and amino acid intakes in relation to prostate cancer risk and mortality—A prospective study in the European Prospective Investigation into Cancer and Nutrition

**DOI:** 10.1002/cam4.5289

**Published:** 2022-09-23

**Authors:** Julie A. Schmidt, Inge Huybrechts, Kim Overvad, Anne Kirstine Eriksen, Anne Tjønneland, Rudolf Kaaks, Verena Katzke, Matthias B. Schulze, Valeria Pala, Carlotta Sacerdote, Rosario Tumino, Bas Bueno‐de‐Mesquita, Maria‐Jose Sánchez, José M. Huerta, Aurelio Barricarte, Pilar Amiano, Antonio Agudo, Anders Bjartell, Tanja Stocks, Elin Thysell, Maria Wennberg, Elisabete Weiderpass, Ruth C. Travis, Timothy J. Key, Aurora Perez‐Cornago

**Affiliations:** ^1^ Cancer Epidemiology Unit, Nuffield Department of Population Health University of Oxford Oxford UK; ^2^ Department of Clinical Epidemiology, Department of Clinical Medicine Aarhus University Hospital and Aarhus University Aarhus N Denmark; ^3^ International Agency for Research on Cancer Lyon France; ^4^ Department of Public Health Aarhus University Aarhus Denmark; ^5^ Danish Cancer Society Research Center Copenhagen Denmark; ^6^ Department of Public Health University of Copenhagen Copenhagen Denmark; ^7^ Division of Cancer Epidemiology, German Cancer Research Center (DKFZ) Heidelberg Germany; ^8^ Department of Molecular Epidemiology German Institute of Human Nutrition Potsdam‐Rehbruecke Nuthetal Germany; ^9^ Institute of Nutritional Science, University of Potsdam Potsdam Germany; ^10^ Epidemiology and Prevention Unit Fondazione IRCCS Istituto Nazionale dei Tumori di Milano Milan Italy; ^11^ Unit of Cancer Epidemiology Città della Salute e della Scienza University‐Hospital Turin Italy; ^12^ Hyblean Association for Epidemiological Research, AIRE ONLUS Ragusa Italy; ^13^ Former senior scientist, Centre for Nutrition, Prevention and Health Services National Institute for Public Health and the Environment (RIVM) Bilthoven The Netherlands; ^14^ Escuela Andaluza de Salud Pública (EASP) Granada Spain; ^15^ Instituto de Investigación Biosanitaria ibs.GRANADA Granada Spain; ^16^ Centro de Investigación Biomédica en Red de Epidemiología y Salud Pública (CIBERESP) Madrid Spain; ^17^ Department of Preventive Medicine and Public Health University of Granada Granada Spain; ^18^ Department of Epidemiology Murcia Regional Health Council, IMIB‐Arrixaca Murcia Spain; ^19^ Instituto de Salud Pública de Navarra Pamplona Spain; ^20^ Ministry of Health of the Basque Government, Sub Directorate for Public Health and Addictions of Gipuzkoa San Sebastian Spain; ^21^ Biodonostia Health Research Institute Epidemiology of Chronic and Communicable Diseases Group San Sebastián Spain; ^22^ Unit of Nutrition and Cancer Catalan Institute of Oncology ‐ ICO L'Hospitalet de Llobregat Spain; ^23^ Nutrition and Cancer Group Epidemiology, Public Health, Cancer Prevention and Palliative Care Program; Bellvitge Biomedical Research Institute—IDIBELL L'Hospitalet de Llobregat Spain; ^24^ Department of Translational Medicine, Medical Faculty Lund University Malmö Sweden; ^25^ Department of Clinical Sciences Lund Lund University Lund Sweden; ^26^ Department of Medical Biosciences Pathology, Umeå University Umeå Sweden; ^27^ Department of Public Health and Clinical Medicine, Section of Sustainable Health Umeå University Umeå Sweden; ^28^ International Agency for Research on Cancer, World Health Organization Lyon France

**Keywords:** dietary amino acid intakes, dietary protein intakes, prostate cancer incidence, prostate cancer mortality, tumour subtypes

## Abstract

**Background:**

The association between protein intake and prostate cancer risk remains unclear.

**Aims:**

To prospectively investigate the associations of dietary intakes of total protein, protein from different dietary sources, and amino acids with prostate cancer risk and mortality.

**Methods:**

In 131,425 men from the European Prospective Investigation into Cancer and Nutrition, protein and amino acid intakes were estimated using validated dietary questionnaires. Multivariable‐adjusted Cox regression models were used to estimate hazard ratios (HRs) and 95% confidence intervals (CIs).

**Results:**

During a mean follow‐up of 14.2 years, 6939 men were diagnosed with prostate cancer and 914 died of the disease. Dairy protein was positively associated with overall prostate cancer risk in the three highest fifths compared to the lowest (HR_Q3_=1.14 (95% CI 1.05–1.23); HR_Q_4=1.09 (1.01–1.18); HR_Q5_=1.10 (1.02–1.19)); similar results were observed for yogurt protein (HR_Q3_=1.14 (1.05–1.24); HR_Q4_=1.09 (1.01–1.18); HR_Q5_=1.12 (1.04–1.21)). For egg protein intake and prostate cancer mortality, no association was observed by fifths, but there was suggestive evidence of a positive association in the analysis per standard deviation increment. There was no strong evidence of associations with different tumour subtypes.

**Discussion:**

Considering the weak associations and many tests, the results must be interpreted with caution.

**Conclusion:**

This study does not provide strong evidence for an association of intakes of total protein, protein from different dietary sources or amino acids with prostate cancer risk or mortality. However, our results may suggest some weak positive associations, which need to be confirmed in large‐scale, pooled analyses of prospective data.

## Graphical abstract

In this large, prospective cohort study, we found no strong associations of protein and amino acid intakes with prostate cancer incidence or mortality. Nonetheless, the results suggested weak positive associations for dairy and yogurt protein with prostate cancer risk, and possibly for egg protein with prostate cancer death. These results deserve further investigation in large‐scale, pooled analyses aimed at clarifying these associations.

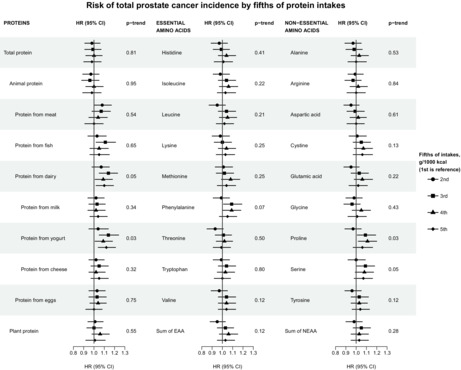

## 1 INTRODUCTION

Prostate cancer is, after lung cancer, the second most common malignancy in men worldwide,^1^ and yet little is known about modifiable risk factors. High circulating concentrations of insulin growth factor‐I (IGF‐I) is a risk factor for prostate cancer,^2^, ^3^ and some evidence indicates that proteins in the diet, especially from dairy products, may be related to higher circulating IGF‐I concentrations.^4^, ^5^, ^6^, ^7^, ^8^, ^9^, ^10^, ^11^, ^12^ Moreover, there is some evidence that supports an association of dairy products with prostate cancer risk, but fewer data on protein from dairy products are available,^13^ and the evidence is still not conclusive.^14^, ^15^

Differences in amino acid composition of protein‐rich foods might partly explain the different associations of protein from different sources with circulating IGF‐I,^16^ and the possible association of dairy protein intake with prostate cancer risk.^13^ A potential role of amino acids in prostate cancer risk is also supported by experimental studies. Intake of essential amino acids stimulates IGF‐I production in rodents, which via activation of mammalian target of rapamycin complex 1 (mTORC1) leads to increased cell proliferation and decreased autophagy and apoptosis.^17^, ^18^, ^19^ Moreover, branched‐chain amino acids, especially leucine, directly activate mTORC1.^19^, ^20^ Finally, cell studies suggest that higher levels of other amino acids, such as arginine, glutamine, glycine, serine and tryptophan may be involved in pathways leading to key processes in cancer development and progression, including proliferation, angiogenesis, cell migration, and metastasis.^21^ However, epidemiological evidence on the associations between amino acid intakes and prostate cancer risk and mortality is lacking.

Therefore, we aimed to investigate the prospective associations of intakes of total protein, protein from ten food groups, 18 amino acids and the sums of essential and non‐essential amino acids with risk of prostate cancer overall, by tumour subtypes, and prostate cancer‐specific mortality in the European Prospective Investigation into Cancer and Nutrition (EPIC).

## 2 MATERIALS AND METHODS

### 2.1 Study population and design

EPIC is a multi‐centre prospective cohort study investigating the role of diet and lifestyle factors in cancer and other diseases in adult men and women. A total of 153,426 men were recruited mostly from the general population between 1992 and 2000 from 19 centres in eight countries (Denmark, Germany, Greece, Italy, Netherlands, Spain, Sweden, and United Kingdom). Details of recruitment and study design have been described in detail elsewhere.^22^ All participants gave written informed consent to participate in the EPIC cohort and approval for the study was obtained from the Internal Review Board of the International Agency for Research on Cancer (IARC), Lyon, France, and the local ethics committees in the participating centres.

We excluded men who were diagnosed with cancer at recruitment (except non‐melanoma skin cancer; *n* = 3972), those with no follow‐up information (*n* = 1433) or date of prostate cancer diagnosis (*n* = 14), men younger than 20 years at recruitment (*n* = 2), men with no non‐dietary or dietary data, or those with extreme energy intake in relation to estimated energy requirement (*n* = 5766),^23^ and men recruited in Greece (*n* = 10,814, because data sharing with Greece was not possible at the time of writing). This left 131,425 men for the current analysis.

### 2.2 Dietary intake and co‐variates

At baseline, participants provided detailed information about their diet, anthropometry, lifestyle, sociodemographic characteristics, and medical history.^22^ Information about food consumption over the past 12 months was collected using validated centre‐specific food frequency questionnaires (FFQs) or diet histories, as previously described.^22^, ^24^ To correct for measurement error between the study centres, dietary intakes for all participants were calibrated using a single standardised, computer‐assisted 24‐hour dietary recall, collected in an 8% representative sample of the cohort on average 1.4 years after recruitment.^25^, ^26^

Intakes of total protein and protein from ten food groups were estimated using the EPIC Nutrient Database (ENDB).^27^ We included total protein, animal protein, protein from meat and meat products (i.e. red meat, processed meat, and poultry combined), protein from fish and fish products (i.e. fish, crustaceans, molluscs, and fish products combined), protein from dairy products (i.e. milk beverages, milk, yogurt, and cheese combined) and each of the latter three dairy subtypes separately, protein from eggs and egg products, and plant protein (calculated as total protein minus animal protein). For animal protein, protein from eggs and plant protein data were not available from recruitment centre Umeå, Sweden. The estimates of total protein intakes in ENDB have been validated using 24‐h urinary nitrogen collected a few days to 5 years after the dietary assessment; in men relatively good agreement between centre means of total nitrogen intake estimated from FFQs and urinary nitrogen were reported (the ratio between mean nitrogen from FFQ and mean urinary nitrogen ranged from 0.75 to 0.90 between the EPIC study centres).^28^

Individual dietary amino acids have been added to the list of nutrients in the ENDB via matching to the the U.S. nutrient database (USNDB, National Nutrient Database for Standard Reference of the U.S. Department of Agriculture (USDA)) using a standardised procedure (Supplementary methods).^29^, ^30^ This matching procedure produced estimates of protein and energy intakes comparable to the validated estimates in ENDB (weighted kappa = 0.84 and 0.89, respectively).^29^ While the estimates of individual amino acid intakes could not be compared with available data in ENDB, the estimates have been compared to those previously estimated independently using a separate protocol in the EPIC‐Oxford centre with very high correlations (*r* ≥ 0.90 for all amino acids).^30^, ^31^

We excluded amino acids for which the mean intake was negligible (i.e. <0.05 g/1000 kcal; *n* = 1, hydroxyproline). The sums of essential amino acids (histidine, isoleucine, leucine, lysine, methionine, phenylalanine, threonine, tryptophan, and valine) and non‐essential amino acids (alanine, arginine, aspartic acid, cysteine, glutamic acid, glycine, proline, serine, and tyrosine) were calculated.

### 2.3 Follow‐up

Follow‐up started on the day of recruitment and ended between January 2011 in Germany and December 2013 in Sweden. Information on cancer incidence, tumour subtypes and vital status was mainly obtained via record linkage to regional and national cancer registries. In Germany a combination of methods was used, including health insurance records, cancer and pathology registries and active follow‐up; self‐reported incident cancers were verified through medical records.

Prostate cancer incidence was defined as code C61 in the 10th revision of the International Statistical Classification of Diseases and Related Health Problems (ICD‐10; *n* = 6939). Prostate cancer mortality was defined as prostate cancer listed as the underlying cause of death on the death certificate (*n* = 914). Histological grade of the tumour was stratified as low‐intermediate grade (Gleason score <8 or coded as well, moderately or poorly differentiated tumours, *n* = 3704) and high grade disease (Gleason score ≥8 or grade coded as undifferentiated tumours, *n* = 724), respectively. The stage of prostate cancer was categorised as localised (tumour‐node‐metastasis [TNM] system score of ≤T_2_ and N_0/x_ and M_0_, or stage coded as localised, *n* = 2606), or advanced (TNM score of T_3‐4_ and/or N_1‐3_ and/or M_1_, or stage coded as advanced, *n* = 1368).

### 2.4 Statistical analysis

Intakes of amino acids and protein were expressed as g/1000 kcal in the main analyses. The mean, standard deviation (SD), and the 25th, 50th and 75th percentiles were tabulated for the exposure variables. Baseline characteristics of the participants were summarised as means (SD) or *n* (%) for continuous and categorical variables, respectively, by fifths of total protein intake.

Cox proportional hazards regression models were used to estimate hazard ratios (HRs) and 95% confidence intervals (CIs) of prostate cancer incidence and mortality for fifths of observed intakes (g/1000 kcal). To test for potential linear trends across the fifths (p_trend_), we used the median values of the fifths as continuous variables in the regression analyses. Additionally, we estimated HRs, 95% CIs, and *p*‐values for a one SD increment (p) in observed and calibrated intakes, although these results are reported only in the supplementary materials because the analyses by fifths suggested some non‐linear associations. Age was used as the underlying time variable in all models; entry time was age at recruitment and exit time was age at censoring, that is, cancer diagnosis, death, last known contact, emigration, or end of the follow‐up, whichever occurred first. All models were stratified by study centre and age at recruitment (<50, 50–54, 55–59, 60–64, 65–69 and ≥70 years) and additionally adjusted for a priori selected confounding factors: baseline body mass index (BMI; <22.5, 22.5–24.9, 25–29.9, ≥30 kg/m^2^, unknown [0.8%]), height (<170, 170–174, 175–179, ≥180 cm, unknown [0.5%]), smoking status (never, former, current, unknown [1.0%]), physical activity (inactive, moderately inactive, moderately active, active, unknown [2.3%]),^32^ educational level (no degree or equivalent, degree or equivalent, unknown [2.9%]), marital status (married or cohabiting, not married or cohabiting, unknown [33.5%]), prevalent diabetes (no, yes, unknown [2.4%]), and energy intake (fifths of the distribution of observed and calibrated energy intakes as appropriate; ENDB for protein variables and USNDB for amino acids). The proportional hazards assumption was checked visually using log–log plots and tested based on Schoenfeld residuals.

Similar Cox regression models were fitted separately for different tumour characteristics (low‐intermediate and high grade, and localised and advanced stage) and follow‐up time (<5 years and ≥5 years).

In sensitivity analyses, the associations between protein intakes and total prostate cancer incidence were estimated with intakes expressed as (i) g/1000 kcal without energy adjustment, (ii) g/d with energy adjustment, and (iii) g/d without energy adjustment.

All tests for statistical significance were two sided. Conventional *p*‐values are shown in order not to miss modest associations, but results were interpreted in the light of multiple testing. To account for multiple testing while allowing for correlation between the 30 exposure variables, we estimated the effective number of independent tests to be nine, using principal component analysis of the exposure variables^33^, ^34^; the first nine principal components explained 99% of the total variation in the data. The statistical significance level after correction for multiple testing was then set to 0.05/9 = 0.0056.

The numbers of participants and cases by fifths of intake are provided in Table S1. All analyses were performed in Stata versions 16 and 17 (Stata Corp LP, College Station, Texas, US).

## 3 RESULTS

After an average follow‐up time of 14.2 years, 6939 men were diagnosed with prostate cancer, including 724 with high grade and 1368 with advanced stage disease; the average age at diagnosis was 68.4 (SD = 6.6) years. The mean follow‐up time for prostate cancer death was 16.2 years, during which 914 men died from prostate cancer, at a mean age of 73.7 (SD = 7.5) years.

### 3.1 Baseline characteristics

Men were on average 52.2 (SD = 9.9) years old at recruitment (Table 1). Men who consumed a larger proportion of their energy intake from protein were on average older at recruitment, had a higher BMI, had a lower daily energy intake, were more likely to smoke, had less formal education, were more likely not to have reported marital status, and were more likely to have diabetes than men in the lowest fifth of energy intake from protein; albeit these differences were all small, except for marital status.

**TABLE 1:** Baseline characteristics by fifths of total protein intake in 131,425 male EPIC participants

	Total protein intake, g/1000 kcal			
	Bottom fifth	Middle fifth	Top fifth	Total sample
	≤34.5	38.3–41.9	≥46.5	10.6–98.9
Number of men	*N* = 26,285	*N* = 26,285	*N* = 26,285	*N* = 131,425
Age at recruitment, years, mean (SD)	51.0 (11.0)	52.2 (9.9)	53.4 (8.6)	52.2 (9.9)
Body mass index, kg/m^2^, mean (SD)	25.5 (3.5)	26.3 (3.5)	27.5 (3.7)	26.4 (3.6)
Height, cm, mean (SD)	176.3 (6.9)	175.4 (7.2)	173.3 (7.4)	175.1 (7.2)
Energy intake, kcal/day, mean (SD)	2473 (709)	2447 (650)	2277 (619)	2417 (662)
Smoking status, *n* (%)				
Never	9946 (37.8%)	8788 (33.4%)	7827 (29.8%)	44,210 (33.6%)
Former	9140 (34.8%)	9817 (37.3%)	9857 (37.5%)	48,280 (36.7%)
Current	6927 (26.4%)	7399 (28.1%)	8324 (31.7%)	37,562 (28.6%)
Unknown	272 (1.0%)	281 (1.1%)	277 (1.1%)	1373 (1.0%)
Physical activity, *n* (%)				
Inactive	4763 (18.1%)	4324 (16.5%)	5330 (20.3%)	23,075 (17.6%)
Moderately inactive	8306 (31.6%)	8172 (31.1%)	7879 (30.0%)	40,646 (30.9%)
Moderately active	6514 (24.8%)	6345 (24.1%)	6142 (23.4%)	31,679 (24.1%)
Active	6254 (23.8%)	6728 (25.6%)	6399 (24.3%)	32,952 (25.1%)
Unknown	448 (1.7%)	716 (2.7%)	535 (2.0%)	3073 (2.3%)
Level of education, *n* (%)				
No degree	17,090 (65.0%)	18,463 (70.2%)	19,557 (74.4%)	92,073 (70.1%)
Degree	8277 (31.5%)	7142 (27.2%)	5859 (22.3%)	35,522 (27.0%)
Unknown	918 (3.5%)	680 (2.6%)	869 (3.3%)	3830 (2.9%)
Marital status, *n* (%)				
Married	17,818 (67.8%)	14,662 (55.8%)	8639 (32.9%)	69,819 (53.1%)
Not married	5474 (20.8%)	3446 (13.1%)	1938 (7.4%)	17,649 (13.4%)
Unknown	2993 (11.4%)	8177 (31.1%)	15,708 (59.8%)	43,957 (33.4%)
Diabetes, *n* (%)				
No	25,209 (95.9%)	24,971 (95.0%)	23,926 (91.0%)	123,987 (94.3%)
Yes	406 (1.5%)	683 (2.6%)	1816 (6.9%)	4332 (3.3%)
Unknown	670 (2.5%)	631 (2.4%)	543 (2.1%)	3106 (2.4%)

The distributions of protein and amino acid intakes are shown in Table S2. The mean observed intake of total protein was 41 (SD = 7) g/1000 kcal, while the mean intakes from animal and plant proteins were 26 (SD = 8) and 15 (SD = 4) g/1000 kcal, respectively. Among the protein sources, yogurt and eggs contributed the least to total protein intake (both mean = 1 and SD = 1 g/1000 kcal). The highest amino acid intake was for glutamic acid (mean = 6 and SD = 1 g/1000 kcal), while the lowest was for tryptophan and cysteine (both mean = 0.4 and SD = 0.1 g/1000 kcal).

### 3.2 Risk of total prostate cancer and prostate cancer death

**FIGURE 1:**
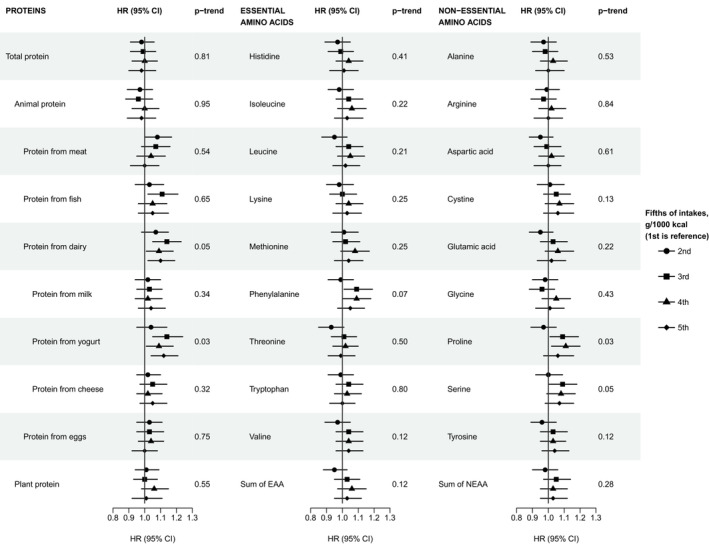
Risk of total prostate cancer incidence by fifths of protein and amino acid intakes in 131,425 male EPIC participants, including 6939 cases. All analyses were stratified for centre and age at entry and adjusted for baseline values of BMI, height, smoking status, physical activity, educational level, marital status, prevalent diabetes, and energy intake. For animal protein, protein from eggs and plant protein data were missing for the recruitment centre Umeå, Sweden; there was a total of 119,383 men in the analyses for these exposures, including 6297 incident cases. Full details of the number of participants and cases by fifth of intakes are shown in Table S1. HRs and 95% CIs for total prostate cancer incidence are shown in Table S3. p_trend_ was calculated by rescoring the fifths with their median values. Abbreviations: CI, confidence intervals; EAA, essential amino acids; HR, Hazard ratio; NEAA, non‐essential amino acids.

We found no strong evidence for associations of protein or amino acids with total prostate cancer risk, as risk estimates were relatively close to 1 and no results were statistically significant after correction for multiple testing. Nonetheless, our results may suggest that men with intermediate and high intakes of protein from total dairy products and from the subtype yogurt have a higher risk of total prostate cancer compared to those with the lowest intakes (Figure 1 and Table S3). For dairy protein, the HRs (95% CIs) in the second to top fifths compared to the bottom fifth were 1.07 (0.98, 1.15), 1.14 (1.05, 1.23), 1.09 (1.01, 1.18), 1.10 (1.02, 1.19; p_trend_ = 0.05), respectively. For protein from yogurt, the corresponding estimates were 1.04 (0.95, 1.14), 1.14 (1.05, 1.24), 1.09 (1.01, 1.18), 1.12 (1.04, 1.21; p_trend_ = 0.03). In contrast, protein intakes from milk and cheese were not associated with risk of prostate cancer, nor was protein from other food sources. For the amino acids, weak positive associations with prostate cancer risk were suggested for the essential amino acid phenylalanine, and the non‐essential amino acids proline and serine (Figure 1 and Table S3). For proline, the HRs (95% CIs) in the second to top fifths compared to the first were 0.97 (0.89, 1.05), 1.09 (1.01, 1.19), 1.11 (1.02, 1.20) and 1.06 (0.97, 1.16; p_trend_ = 0.03), respectively. The results for phenylalanine and serine were similar to those for proline; risk estimates in the second to top fifth compared to the first were 0.99 (0.91, 1.07), 1.09 (1.01, 1.19), 1.09 (1.00, 1.18), 1.05 (0.97, 1.14; p_trend_ = 0.07), and 1.00 (0.92, 1.09), 1.09 (1.00, 1.18), 1.08 (0.99, 1.17) and 1.07 (0.98, 1.16; p_trend_ = 0.05), respectively. When modelling protein and amino acids intakes (both observed and calibrated) as linear variables for a SD increment, no associations were observed (Table S3).

**FIGURE 2:**
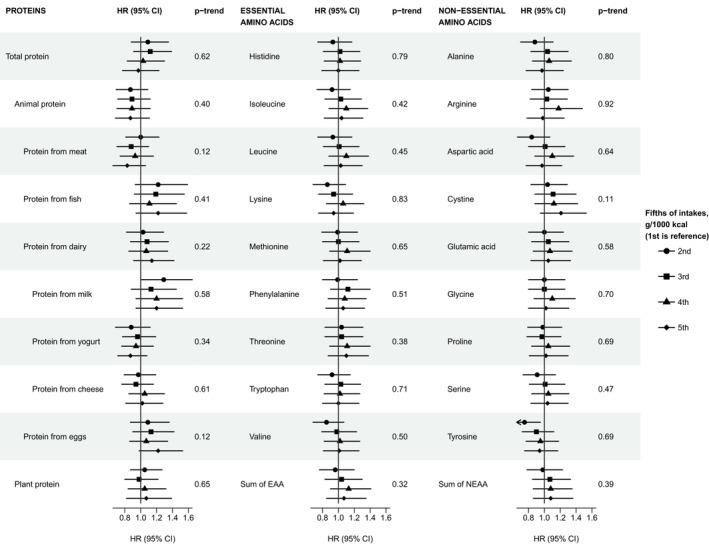
Risk of prostate cancer mortality by fifths of protein anamino acid intakes in 131,425 male EPIC participants, including 914 deaths. All analyses were stratified for centre and age at entry and adjusted for baseline values of BMI, height, smoking status, physical activity, educational level, marital status, prevalent diabetes, and energy intake. For animal protein, protein from eggs and plant protein data were missing for the recruitment centre Umeå, Sweden; there was a total of 119,383 men in the analyses for these exposures, including 860 deaths. Full details of the number of participants and cases by fifth of intakes are shown in Table S1. HRs and 95% CIs for prostate cancer mortality are shown in Table S4. p_trend_ was calculated by rescoring the fifths with their median values. Abbreviations: CI, confidence intervals; EAA, essential amino acids; HR, Hazard ratio; NEAA, non‐essential amino acids.

For prostate cancer death, we observed no strong associations with protein or amino acid intakes, but the results suggested that men who consumed more protein from eggs might be at a higher risk of dying from prostate cancer (Figure 2 and Table S4). The HRs (95% CIs) in the second to the top fifths compared to the lowest fifth were 1.09 (0.87, 1.36), 1.13 (0.90, 1.42), 1.07 (0.86, 1.34) and 1.22 (0.98, 1.53; p_trend_ = 0.1), respectively. Moreover, in the continuous models of observed and calibrated intakes, the HRs per SD higher intakes of protein from eggs were 1.07 (1.10, 1.14; *p* = 0.03) and 1.11 (1.03, 1.20; *p* = 0.005), respectively (Table S4). None of the results, besides the association for the calibrated intake of protein from egg, were statistically significant after correction for multiple testing.

### 3.3 Subgroup analyses by tumour subtypes and follow‐up time

There was little evidence of associations with high grade prostate cancer, except for an inverse trend for total protein intake and risk of high grade prostate cancer (HR_Q5_ = 0.76 [0.59–1.00], p_trend_ = 0.04; Tables 2 and S5). Results for low‐intermediate grade were generally in line with those for total prostate cancer risk (Table S5).

**TABLE 2:** Risk of high grade prostate cancer by fifths of intakes of proteins and amino acids in 128,914 male EPIC participants, including 724 high grade cases^a^ ^,^ ^b^ ^,^ ^c^

Intakes	Fifths of intakes, g/1000 kcal					
	1	2	3	4	5	p_trend_ ^d^
	HR (95% CI)	HR (95% CI)	HR (95% CI)	HR (95% CI)	HR (95% CI)	
Protein from food groups						
Total protein	1 (Ref)	0.93 (0.73, 1.18)	0.95 (0.75, 1.21)	0.87 (0.68, 1.12)	0.76 (0.59, 1.00)	0.04
Animal protein	1 (Ref)	0.82 (0.62, 1.08)	0.92 (0.70, 1.20)	0.94 (0.71, 1.22)	0.80 (0.60, 1.05)	0.2
Protein from meat	1 (Ref)	1.02 (0.80, 1.30)	1.02 (0.79, 1.31)	0.96 (0.74, 1.25)	0.92 (0.70, 1.21)	0.4
Protein from fish	1 (Ref)	1.07 (0.81, 1.43)	1.22 (0.93, 1.61)	1.14 (0.86, 1.52)	0.90 (0.66, 1.22)	0.1
Protein from dairy products	1 (Ref)	1.03 (0.81, 1.31)	0.99 (0.78, 1.27)	1.05 (0.82, 1.33)	1.02 (0.79, 1.30)	0.9
Protein from milk	1 (Ref)	1.09 (0.85, 1.41)	1.01 (0.78, 1.30)	0.89 (0.68, 1.15)	0.91 (0.70, 1.18)	0.2
Protein from yogurt	1 (Ref)	1.30 (0.98, 1.72)	1.24 (0.96, 1.60)	1.12 (0.86, 1.45)	1.19 (0.92, 1.53)	0.9
Protein from cheese	1 (Ref)	0.97 (0.77, 1.22)	1.07 (0.85, 1.35)	1.02 (0.80, 1.31)	1.11 (0.86, 1.43)	0.4
Protein from eggs	1 (Ref)	1.02 (0.80, 1.31)	0.99 (0.76, 1.28)	0.99 (0.77, 1.27)	0.92 (0.71, 1.19)	0.4
Plant protein	1 (Ref)	0.94 (0.74, 1.19)	0.92 (0.72, 1.19)	1.19 (0.92, 1.53)	0.98 (0.73, 1.32)	0.7
Amino acids						
Essential amino acids						
Histidine	1 (Ref)	1.01 (0.79, 1.29)	1.08 (0.85, 1.38)	1.05 (0.82, 1.35)	0.84 (0.65, 1.10)	0.2
Isoleucine	1 (Ref)	0.95 (0.74, 1.22)	1.10 (0.86, 1.41)	1.00 (0.78, 1.29)	0.88 (0.68, 1.15)	0.4
Leucine	1 (Ref)	0.98 (0.77, 1.26)	1.10 (0.86, 1.41)	1.04 (0.81, 1.33)	0.89 (0.68, 1.16)	0.4
Lysine	1 (Ref)	0.81 (0.63, 1.04)	1.03 (0.81, 1.31)	0.84 (0.65, 1.08)	0.80 (0.62, 1.04)	0.1
Methionine	1 (Ref)	0.98 (0.76, 1.25)	1.07 (0.84, 1.37)	1.01 (0.79, 1.30)	0.86 (0.66, 1.12)	0.3
Phenylalanine	1 (Ref)	0.97 (0.76, 1.25)	1.07 (0.84, 1.37)	1.06 (0.83, 1.36)	0.91 (0.70, 1.18)	0.6
Threonine	1 (Ref)	0.90 (0.70, 1.16)	1.09 (0.86, 1.39)	0.90 (0.70, 1.16)	0.87 (0.67, 1.13)	0.3
Tryptophan	1 (Ref)	0.82 (0.64, 1.05)	1.02 (0.81, 1.29)	0.90 (0.70, 1.14)	0.82 (0.64, 1.06)	0.2
Valine	1 (Ref)	1.00 (0.78, 1.28)	1.08 (0.84, 1.38)	1.03 (0.80, 1.32)	0.89 (0.69, 1.16)	0.4
Sum of essential amino acid	1 (Ref)	1.03 (0.80, 1.32)	1.09 (0.85, 1.40)	1.12 (0.87, 1.44)	0.90 (0.69, 1.17)	0.5
Non‐essential amino acids						
Alanine	1 (Ref)	1.03 (0.80, 1.32)	1.01 (0.78, 1.30)	0.96 (0.74, 1.25)	0.91 (0.69, 1.19)	0.4
Arginine	1 (Ref)	1.15 (0.90, 1.46)	1.00 (0.78, 1.29)	1.06 (0.82, 1.37)	0.89 (0.68, 1.17)	0.3
Aspartic acid	1 (Ref)	0.90 (0.70, 1.16)	0.96 (0.75, 1.23)	0.89 (0.69, 1.15)	0.81 (0.63, 1.06)	0.1
Cystine	1 (Ref)	1.15 (0.89, 1.49)	1.27 (0.98, 1.65)	1.12 (0.85, 1.48)	1.11 (0.84, 1.47)	0.7
Glutamic acid	1 (Ref)	0.99 (0.77, 1.26)	1.06 (0.83, 1.36)	1.04 (0.80, 1.34)	0.93 (0.71, 1.22)	0.7
Glycine	1 (Ref)	1.02 (0.80, 1.31)	1.00 (0.77, 1.29)	1.11 (0.86, 1.44)	0.92 (0.70, 1.22)	0.7
Serine	1 (Ref)	1.01 (0.79, 1.29)	1.06 (0.83, 1.35)	0.99 (0.77, 1.27)	0.92 (0.71, 1.19)	0.4
Tyrosine	1 (Ref)	0.86 (0.67, 1.10)	0.95 (0.75, 1.21)	0.94 (0.74, 1.20)	0.87 (0.68, 1.13)	0.5
Sum of non‐essential amino acids	1 (Ref)	0.91 (0.71, 1.17)	1.04 (0.81, 1.32)	0.95 (0.74, 1.22)	0.90 (0.69, 1.17)	0.5

^a^ All analyses were stratified for centre and age at entry and adjusted for baseline values of BMI, height, smoking status, physical activity, educational level, marital status, prevalent diabetes, and energy intake.

^b^ For animal protein, protein from eggs and plant protein data were missing for the recruitment centre Umeå, Sweden; there was a total of 116,959 men in the analyses for these exposures, including 645 cases.

^c^ The number of participants and cases by fifth of intakes are shown in Table S1.

^d^ p_trend_ was calculated by rescoring the fifths with their median values.

There was no evidence of associations between protein or amino acids and risk for advanced stage prostate cancer (Tables 3 and S6), and the associations with localised prostate cancer were mostly similar to those with total prostate cancer incidence (Table S6).

**TABLE 3:** Risk of advanced stage prostate cancer by fifths of intakes of proteins and amino acids in 128,460 male EPIC participants, including 1368 advanced stage cases^a^ ^,^ ^b^ ^,^ ^c^

Intakes	Fifths of intakes, g/1000 kcal					
	1	2	3	4	5	p_trend_ ^d^
	HR (95% CI)	HR (95% CI)	HR (95% CI)	HR (95% CI)	HR (95% CI)	
Protein from food groups						
Total protein	1 (Ref)	0.92 (0.77, 1.10)	0.96 (0.81, 1.14)	0.83 (0.69, 1.00)	0.89 (0.74, 1.08)	0.2
Animal protein	1 (Ref)	1.03 (0.85, 1.25)	0.94 (0.77, 1.14)	0.91 (0.74, 1.11)	0.97 (0.79, 1.19)	0.5
Protein from meat	1 (Ref)	0.96 (0.80, 1.16)	0.94 (0.78, 1.14)	0.93 (0.77, 1.13)	0.88 (0.72, 1.08)	0.2
Protein from fish	1 (Ref)	1.18 (0.96, 1.44)	1.22 (1.00, 1.50)	1.03 (0.84, 1.27)	1.10 (0.89, 1.37)	0.8
Protein from dairy products	1 (Ref)	0.99 (0.83, 1.18)	1.03 (0.87, 1.23)	1.03 (0.87, 1.23)	1.06 (0.89, 1.26)	0.5
Protein from milk	1 (Ref)	1.04 (0.87, 1.24)	0.95 (0.80, 1.15)	0.93 (0.77, 1.12)	1.00 (0.83, 1.21)	0.8
Protein from yogurt	1 (Ref)	1.12 (0.92, 1.37)	1.15 (0.96, 1.39)	1.10 (0.91, 1.32)	1.08 (0.89, 1.30)	0.9
Protein from cheese	1 (Ref)	1.09 (0.91, 1.29)	1.13 (0.95, 1.35)	1.13 (0.94, 1.36)	1.15 (0.95, 1.39)	0.2
Protein from eggs	1 (Ref)	1.10 (0.92, 1.31)	0.97 (0.81, 1.17)	0.99 (0.83, 1.20)	0.91 (0.75, 1.10)	0.1
Plant protein	1 (Ref)	1.02 (0.87, 1.20)	1.02 (0.86, 1.21)	0.98 (0.81, 1.18)	1.04 (0.84, 1.30)	0.8
Amino acids						
*Essential amino acids*						
Histidine	1 (Ref)	0.94 (0.78, 1.12)	0.98 (0.81, 1.17)	0.98 (0.82, 1.18)	0.92 (0.76, 1.11)	0.5
Isoleucine	1 (Ref)	0.90 (0.75, 1.08)	1.00 (0.84, 1.20)	0.97 (0.81, 1.16)	0.93 (0.77, 1.12)	0.6
Leucine	1 (Ref)	0.94 (0.79, 1.14)	1.00 (0.83, 1.20)	1.04 (0.87, 1.25)	0.96 (0.79, 1.16)	0.9
Lysine	1 (Ref)	0.96 (0.80, 1.16)	0.97 (0.81, 1.16)	0.98 (0.81, 1.18)	0.95 (0.78, 1.15)	0.7
Methionine	1 (Ref)	0.99 (0.82, 1.20)	1.06 (0.88, 1.27)	1.00 (0.83, 1.21)	0.97 (0.80, 1.18)	0.7
Phenylalanine	1 (Ref)	0.95 (0.79, 1.15)	1.05 (0.88, 1.26)	1.10 (0.92, 1.32)	0.96 (0.80, 1.16)	1.0
Threonine	1 (Ref)	0.89 (0.75, 1.07)	0.94 (0.79, 1.13)	0.95 (0.79, 1.14)	0.91 (0.76, 1.10)	0.6
Tryptophan	1 (Ref)	0.90 (0.75, 1.08)	1.04 (0.87, 1.23)	0.93 (0.78, 1.12)	0.92 (0.76, 1.10)	0.5
Valine	1 (Ref)	0.88 (0.73, 1.05)	1.02 (0.85, 1.22)	0.96 (0.80, 1.15)	0.95 (0.79, 1.14)	0.8
Sum of essential amino acid	1 (Ref)	0.98 (0.82, 1.18)	1.01 (0.84, 1.22)	1.04 (0.86, 1.25)	0.97 (0.81, 1.18)	0.9
*Non‐essential amino acids*						
Alanine	1 (Ref)	0.96 (0.80, 1.16)	0.95 (0.79, 1.15)	0.97 (0.80, 1.18)	0.93 (0.76, 1.14)	0.6
Arginine	1 (Ref)	1.06 (0.88, 1.27)	1.01 (0.84, 1.21)	1.04 (0.86, 1.25)	0.98 (0.81, 1.19)	0.7
Aspartic acid	1 (Ref)	0.86 (0.72, 1.04)	0.95 (0.79, 1.14)	0.99 (0.83, 1.19)	0.91 (0.75, 1.10)	0.7
Cystine	1 (Ref)	0.99 (0.81, 1.20)	1.04 (0.85, 1.27)	1.12 (0.92, 1.36)	1.06 (0.86, 1.30)	0.4
Glutamic acid	1 (Ref)	0.83 (0.69, 1.00)	1.06 (0.89, 1.27)	1.01 (0.84, 1.22)	0.95 (0.78, 1.16)	0.8
Glycine	1 (Ref)	1.13 (0.93, 1.37)	1.03 (0.85, 1.26)	1.08 (0.89, 1.32)	1.09 (0.89, 1.34)	0.6
Serine	1 (Ref)	0.87 (0.72, 1.05)	1.05 (0.88, 1.25)	1.03 (0.86, 1.23)	0.94 (0.78, 1.13)	0.9
Tyrosine	1 (Ref)	0.84 (0.70, 1.01)	0.95 (0.79, 1.14)	0.96 (0.80, 1.15)	0.93 (0.77, 1.11)	0.9
Sum of non‐essential amino acids	1 (Ref)	0.92 (0.77, 1.11)	0.97 (0.81, 1.17)	1.03 (0.85, 1.24)	0.96 (0.80, 1.17)	1.0

^a^ All analyses were stratified for centre and age at entry and adjusted for baseline values of BMI, height, smoking status, physical activity, educational level, marital status, prevalent diabetes, and energy intake.

^b^ For animal protein, protein from eggs and plant protein data were missing for the recruitment centre Umeå, Sweden; there was a total of 116,418 men in the analyses for these exposures, including 1282 cases.

^c^ The number of participants and cases by fifth of intakes are shown in Table S1.

^d^ p_trend_ was calculated by rescoring the fifths with their median values.

When stratifying the analyses of prostate cancer incidence by follow‐up time, the risk estimates in the subgroup with five or more years of follow‐up were similar to those in the main analysis (Table S7). This suggests that reverse causation had little impact on the main findings in the full study population.

### 3.4 Sensitivity analyses

Analysing intakes as g/d rather than g/1000 kcal did not materially change the results, neither did excluding energy intake from the regression models (Table S8).

## 4 DISCUSSION

In this large European prospective study, we did not find strong evidence for associations of intakes of total protein, protein from different dietary sources or amino acids with prostate cancer incidence (overall or by tumour subtypes) or prostate cancer‐specific mortality. However, our results may suggest that men who consume more protein from dairy products, including protein from yogurt, are at higher risk of prostate cancer overall, and possibly that men with higher intakes of protein from eggs might be at higher risk of dying from prostate cancer.

Similarly to the current results, a previous analysis in EPIC based on the first 2727 prostate cancer cases also reported a positive association between intakes of dairy protein and prostate cancer risk.^13^ In the current analyses, we were able to add five additional years of follow‐up to the data from the previous analysis and now have 2.5‐fold the number of incident cases (a total of *n* = 6939). Moreover, we extended our analysis to include prostate cancer death and a larger range of dietary exposures, including dairy subtypes and novel data on amino acids. The previous analysis found that dairy protein was associated with a higher risk of high grade prostate cancer, which was not observed in the updated analyses. However, in the current analyses we have used a stricter definition of high grade prostate cancer including only Gleason score ≥8, while the previous analyses also included Gleason score 7 as high grade disease. When looking at protein from different dairy subgroups separately, we found similar results for protein intake from yogurt (but not from milk or cheese) to those observed for total dairy protein. The latest World Cancer Research Fund/American Institute for Cancer Research (WCRF/AICR) report on prostate cancer concluded that there was limited suggestive evidence for a positive association between total dairy products consumption (as a food group as opposed to protein from dairy) and prostate cancer risk; no association was found for yogurt consumption.^15^, ^35^ A more recent publication synthesising information from meta‐analyses and systematic reviews of dairy consumption and prostate cancer risk also reported inconclusive findings.^14^ Thus, further research is needed, especially investigating aggressive tumour subtypes.

A potential mechanism for the possible association between protein from dairy products and prostate cancer risk is through higher circulating IGF‐I, which is an established risk factor for prostate cancer.^2^, ^3^ In observational studies, protein from dairy products has consistently been reported to be positively associated with circulating IGF‐I concentrations,^5^, ^6^, ^7^, ^8^, ^9^ and it has been suggested that the association may be specific to protein from yogurt and milk but not cheese.^9^ There is also some evidence from randomised controlled trials showing that increased consumption of dairy products leads to an increase in circulating IGF‐I.^36^, ^37^ However, we cannot determine if it is protein (or amino acids) in dairy, or possibly other components of dairy products or factors related to dairy consumption which may be responsible for the observed associations. Another component in dairy products which has been hypothesised as a possible mechanism is calcium, but the evidence is inconclusive.^38^

We are not aware of previous studies that have investigated the associations between protein from eggs and prostate cancer mortality. However, the Spearman correlation between intake of eggs (as a food) and protein from eggs (both expressed as g/1000 kcal) is >0.99 in our data, therefore we will here compare our results to those from prospective analyses of consumption of eggs and risk of fatal prostate cancer. The Pooling Project of Prospective Studies of Diet and Cancer, which combined data from ten prospective studies (including 29% of the prostate cancer deaths from the current analysis, in a total of 3199 fatal cases) reported a higher risk of prostate cancer death in men who consumed 25 g of eggs/day or more compared to those who consumed less than 5 g/day (RR = 1.14, 95% CI 1.00, 1.30, p_trend_ = 0.01; 1 egg weighs ~50 g).^39^ Similar results have been reported in a recent small meta‐analysis^40^ and the WCRF/AICR Continuous Update Project.^15^ Both included the same four prospective studies (with 609 prostate cancer deaths), one of which^41^ was also included in the Pooling Project of Prospective Studies of Diet and Cancer.^39^

It is possible that the observed positive association between protein from eggs and prostate cancer mortality is driven by factors other than protein in eggs. For example, eggs have a high content of both cholesterol and choline,^39^, ^40^ which might be related to prostate cancer development and progression,^42^, ^43^ although, the evidence from prospective studies is limited.^44^ An alternative explanation for this association might be that some health‐conscious men, who may be diagnosed with prostate cancer earlier potentially leading to a better prognosis, consume fewer eggs due to the previous wide‐spread recommendation to restrict egg intake for blood cholesterol level control.^45^ However, we did not observe heterogeneity by stage for the association between protein from eggs and risk, which might be expected if this was the case.

Our results do not provide strong evidence for an association between amino acid intakes and prostate cancer risk or mortality, although they might suggest weak positive associations of phenylalanine, proline, and serine with risk of total prostate cancer incidence. To the best of our knowledge, the only previous epidemiological research on intake of amino acids and prostate cancer risk is focused on methionine as part of one‐carbon metabolism and does not suggest an association of methionine intake with prostate cancer incidence or mortality.^46^, ^47^, ^48^ Thus, our finding needs to be confirmed in other studies.

We are not aware of other studies reporting results for total protein intake and high grade prostate cancer. Given the lack of evidence and the limited number of high grade cases in our analysis (*n* = 724), the observed inverse association needs to be studied further.

The main strengths of this study are the prospective design and the large, well‐characterised cohort with detailed data on dietary intake (including novel data on amino acid intakes), lifestyle, and the large number of prostate cancer cases. These features allowed us to investigate the associations of intakes of total protein, protein from several dietary sources and 18 amino acids with risk of prostate cancer overall, prostate cancer death, and by tumour subtypes, while adjusting for potential confounding factors. The study further benefited from the long follow‐up and reliable assessment of cancer diagnoses via cancer registries or data verified using medical records.

This study also has several limitations. Firstly, unmeasured and residual confounding from, for example, prostate‐specific antigen testing, and due to missing values in some covariates, cannot be ruled out. Secondly, the dietary data have some weaknesses. We used a single dietary questionnaire to estimate intakes of amino acids and protein and were thus not able to account for dietary changes during follow‐up. This will have led to some miss‐classification of usual intakes over the follow‐up period. Although the questionnaires were validated, and we calibrated the dietary intakes, random measurement error is inevitable and our results are likely to be biased towards the null. Secondly, for foods that may not be consumed daily, such as eggs, calibration using a single 24‐h recall might not be adequate. Thus, results from the model of calibrated intake of egg as a continuous variable should be interpreted very cautiously. Thirdly, some of our findings may be due to chance because of the large number of tests conducted. Finally, we were not able to study the use of protein supplementation, e.g. protein powder used after workouts. While such supplementation is likely uncommon in our study population and thus unlikely to affect our results, the association of protein supplementation with risk of prostate cancer deserves investigation.

In conclusion, the current analysis did not provide strong support for any associations of intakes of total protein, protein from different dietary sources or amino acids with prostate cancer risk or mortality. While a role of chance cannot be ruled out, our results might suggest that men who consume more protein from dairy products and from yogurt might be at higher risk of prostate cancer. Moreover, our results suggest that men who eat more egg protein might be at higher risk of dying from the disease. For firmer conclusions to be drawn, pooled data from large‐scale prospective studies, ideally with repeat measures of dietary intakes, data on tumour subtypes, and with up‐to‐date outcome data are needed.

## AUTHOR CONTRIBUTIONS

**Julie A. Schmidt:** Conceptualization (equal); data curation (equal); formal analysis (lead); funding acquisition (equal); investigation (lead); methodology (equal); project administration (lead); visualization (lead); writing – original draft (lead); writing – review and editing (lead). **Inge Huybrechts:** Data curation (equal); investigation (equal); methodology (supporting); resources (equal); writing – review and editing (equal). **Kim Overvad:** Investigation (equal); methodology (supporting); resources (equal); writing – review and editing (equal). **Anne Kirstine Eriksen:** Investigation (equal); methodology (supporting); resources (equal); writing – review and editing (equal). **Anne Tjonneland:** Investigation (equal); methodology (supporting); resources (equal); writing – review and editing (equal). **Rudolf Kaaks:** Investigation (equal); methodology (supporting); resources (equal); writing – review and editing (equal). **Verena Katzke:** Investigation (equal); methodology (supporting); resources (equal); writing – review and editing (equal). **Matthias B Schulze:** Investigation (equal); methodology (supporting); resources (equal); writing – review and editing (equal). **Valeria Pala:** Investigation (equal); methodology (supporting); resources (equal); writing – review and editing (equal). **Carlotta Sacerdote:** Investigation (equal); methodology (supporting); resources (equal); writing – review and editing (equal). **Rosario Tumino:** Investigation (equal); methodology (supporting); resources (equal); writing – review and editing (equal). **H Bas Bueno‐de‐Mesquita:** Investigation (equal); methodology (supporting); resources (equal); writing – review and editing (equal). **Maria‐Jose Sanchez:** Investigation (equal); methodology (supporting); resources (equal); writing – review and editing (equal). **Jose Maria Huerta:** Investigation (equal); methodology (supporting); resources (equal); writing – review and editing (equal). **Aurelio Barricarte Gurrea:** Investigation (equal); methodology (supporting); resources (equal); writing – review and editing (equal). **Pilar Amiano:** Investigation (equal); methodology (supporting); resources (equal); writing – review and editing (equal). **Antonio Agudo:** Investigation (equal); methodology (supporting); resources (equal); writing – review and editing (equal). **Anders Bjartell:** Investigation (equal); methodology (supporting); resources (equal); writing – review and editing (equal). **Tanja Stocks:** Investigation (equal); methodology (supporting); resources (equal); writing – review and editing (equal). **Elin Thysell:** Investigation (equal); methodology (supporting); resources (equal); writing – review and editing (equal). **Maria Wennberg:** Investigation (equal); methodology (supporting); resources (equal); writing – review and editing (equal). **Elisabete Weiderpass:** Investigation (equal); methodology (supporting); resources (equal); writing – review and editing (equal). **Ruth Travis:** Funding acquisition (equal); investigation (equal); methodology (equal); project administration (supporting); resources (equal); supervision (equal); visualization (supporting); writing – original draft (supporting); writing – review and editing (equal). **Timothy J. Key:** Conceptualization (equal); funding acquisition (lead); investigation (equal); methodology (equal); project administration (supporting); resources (equal); supervision (equal); writing – original draft (supporting); writing – review and editing (equal). **Aurora Perez‐Cornago:** Conceptualization (equal); data curation (equal); formal analysis (supporting); funding acquisition (equal); investigation (equal); methodology (equal); project administration (supporting); resources (equal); supervision (lead); visualization (supporting); writing – original draft (supporting); writing – review and editing (equal).

## FUNDING INFORMATION

This work was supported by Cancer Research UK (C8221/A29017). The coordination of the European Prospective Investigation into Cancer and Nutrition (EPIC) is financially supported by the International Agency for Research on Cancer and has been supported by the European Commission (DG‐SANCO). The national cohorts are supported by Danish Cancer Society (Denmark); German Cancer Aid, German Cancer Research Center (DKFZ), Federal Ministry of Education and Research (BMBF) (Germany); Associazione Italiana per la Ricerca sul Cancro‐AIRC‐Italy and National Research Council (Italy); Dutch Ministry of Public Health, Welfare and Sports (VWS), Netherlands Cancer Registry (NKR), LK Research Funds, Dutch Prevention Funds, Dutch ZON (Zorg Onderzoek Nederland), World Cancer Research Fund (WCRF), Statistics Netherlands (The Netherlands); Health Research Fund (FIS) ‐ Instituto de Salud Carlos III (ISCIII), Regional Governments of Andalucía, Asturias, Basque Country, Murcia and Navarra, and the Catalan Institute of Oncology ‐ ICO (Spain); Swedish Cancer Society, Swedish Scientific Council, and Regional Government of Skåne and Västerbotten (Sweden); Cancer Research UK (14136 to EPIC‐Norfolk; C570/A16491 to EPIC‐Oxford), Medical Research Council (1000143 to EPIC‐Norfolk, MR/M012190/1 to EPIC‐Oxford) (UK).

## CONFLICT OF INTEREST

The authors declare no conflicts of interest.

## DISCLAIMER

Where authors are identified as personnel of the International Agency for Research on Cancer / World Health Organisation, the authors alone are responsible for the views expressed in this article and they do not necessarily represent the decisions, policy or views of the International Agency for Research on Cancer / World Health Organisation.

## Supporting information


Appendix S1

Table S1
Table S2.
Table S3
**Table S4**.
Table S5
**Table S6**.
Table S7
Table S8

## Data Availability

For information on how to submit an application for gaining access to EPIC data and/or biospecimens, please follow the instructions at http:// epic.iarc.fr/access/index.php
