# Supplementary material for: Protein and amino acid intakes in relation to prostate cancer risk and mortality—A prospective study in the European Prospective Investigation into Cancer and Nutrition
Source: Cancer Med. 2022 Sep 23;12(4):4725–38. doi: 10.1002/cam4.5289 (PMC9972153; doi:10.1002/cam4.5289)
Supplement: Supplementary file 1 — Appendix S1 [file CAM4-12-4725-s002.docx]

# Supplementary methods

## Estimation of amino acid intakes

Amino acid composition of foods is not generally available in the European nutrient tables used in the European Prospective Investigation into Cancer and Nutrition (EPIC) and amino acid intakes were thus until recently not available in the EPIC Nutrient Database (ENDB). However, data are available from (USNDB, National Nutrient Database for Standard Reference of the U.S. Department of Agriculture (USDA)). Thus, food items from the two databases were linked to obtain values for intakes of amino acids for participants in EPIC ^1, 2^. This was done by three dietitians and an experienced database manager using a standardised procedure under supervision of a nutritionist and a food chemist with expertise in this ENDB linking. The USNDB release 26 (October 2013) with 8463 food items was used, with further completion using the 28th release (September 2015) containing 8789 food items ^1^.

To avoid errors, extensive quality control was applied. First, the matching was checked within the team of dietitians. Second, values for 28 nutrients (including total protein and energy) obtained from USNDA were compared to those previously obtained in ENDB using national nutrient tables; the comparison was done using Bland-Altman plots, correlations and weighted kappa ^1^. For total protein, there was excellent agreement between the estimated intakes from ENDB and USNDA, with a correlation coefficient and weighted kappa of 0.97 and 0.84 for the dietary questionnaire data, respectively. Similar results were obtained for energy intakes, (correlation coefficients and weighted kappa of 0.98 and 0.89, respectively) ^1^.

The estimated intakes of amino acid have been compared with amino acids intakes estimated independently by a researcher using a separate protocol in the EPIC-Oxford study centre ^2, 3^. This comparison showed high correlations between the amino acid intakes estimated using the two methods and investigators (correlation coefficients ≥0.90 for all amino acids) ^2^.

**References**

1. Van Puyvelde H, Perez-Cornago A, Casagrande C, Nicolas G, Versele V, Skeie G, B. Schulze M, Johansson I, María Huerta J, Oliverio A, Ricceri F, Halkjær J, Amiano Etxezarreta P, Van Herck K, Weiderpass E, J. Gunter M, Huybrechts I. Comparing Calculated Nutrient Intakes Using Different Food Composition Databases: Results from the European Prospective Investigation into Cancer and Nutrition (EPIC) Cohort. Nutrients 2020; 12: 2906

2. Iguacel I, Perez-Cornago A, Schmidt JA, Van Puyvelde H, Travis R, Casagrande C, Nicolas G, Riboli E, Weiderpass E, Ardanaz E, Barricarte A, Bodén S, Bruno E, Ching-López A, Aune D, Jensen TE, Ericson U, Johansson I, Ma Huerta J, Katzke V, Kühn T, Sacerdote C, Schulze MB, Skeie G, Ramne S, Ward H, Gunter MJ, Huybrechts I. Evaluation of protein and amino acid intake estimates from the EPIC dietary questionnaires and 24-h dietary recalls using different food composition databases. Nutr Metab Cardiovasc Dis 2022; 32: 80-9

3. Schmidt JA, Rinaldi S, Scalbert A, Ferrari P, Achaintre D, Gunter MJ, Appleby PN, Key TJ, Travis RC. Plasma concentrations and intakes of amino acids in male meat-eaters, fish-eaters, vegetarians and vegans: a cross-sectional analysis in the EPIC-Oxford cohort. Eur J Clin Nutr 2016; 70: 306-12
